# A polysaccharide from the dried rhizome of Drynaria fortunei (Kunze) J. Sm. prevents ovariectomized (OVX)‐induced osteoporosis in rats

**DOI:** 10.1111/jcmm.15072

**Published:** 2020-02-17

**Authors:** Xin Sun, Bo Wei, Zhiheng Peng, Xiaru Chen, Qinglong Fu, Chaojun Wang, Jinchang Zhen, Jiecong Sun

**Affiliations:** ^1^ Department of Orthopaedic Surgery Affiliated Hospital of Guangdong Medical University Zhanjiang China

**Keywords:** anti‐osteoporotic effect, Drynaria fortunei polysaccharide, osteoporosis

## Abstract

In the present study, a homogenous polysaccharide (DFPW) was isolated and purified from the dried rhizome of *Drynaria fortunei,* and its protective effect against osteoporosis was investigated in ovariectomized (OVX) rats. Histological analysis indicated that oral administration of DFPW (100 and 400 mg/kg) for 12 weeks significantly improved trabecular bone mass, as demonstrated by the increase in trabecular area, trabecular thickness and its number in OVX rats. Furthermore, the decline of bone mineral density and bone mineral content including Ca, P and Mg induced by OVX was reversed by the DFPW administration. This function was achieved by the decreased levels of the bone turnover markers, such as serum ALP, urinary deoxypyridinoline (DPD), Ca and P excretions. Besides, DFPW improved biomechanical parameters (maximum load, energy, Young's, modulus and maximum stress) to strengthen the hardness and strength femoral diaphysis in OVX rats. These results strongly suggested that DFPW might be a hopeful alternative therapeutics to treat postmenopausal osteoporosis.

## 1 INTRODUCTION

Osteoporosis is a metabolic bone disease, characterized by low bone mass and impaired microarchitecture of bone tissue, which increases bone frailty and susceptibility to the risk of fractures.^1^, ^2^ Now, osteoporosis is widely reckoned as a major public health hazard, with high death rate and medical expenses throughout the world.^3^ In most regions, the incidence of osteoporosis is approximately 2 to 4 times higher in female than in male. The most common one is postmenopausal osteoporosis, which occurs predominantly in postmenopausal women because of dramatic ovarian oestrogen deficiency after menopause.^4^ Current strategy for the design of anti‐osteoporotic drug is based on two options: one is to prevent bone resorption and the other is to stimulate bone formation.^5^ Although administration of oestrogen on onset or after the menopause rescues bone mass and alleviates fracture rate, long‐term oestrogen therapy may run high risk of uterine and breast cancer incidence.^6^ In addition, hormone replacement therapy (HRT) is one of the most popular and effective regime for preventing bone loss and decreasing the incidence of postmenopausal osteoporosis.^7^ However, reports from the Women's Health Initiative Trial suggest that long‐term use of HRT has been related to increased risk of ovarian, breast and endometrial cancers, not to mention many non‐fatal side effects, such as thromboembolic events and vaginal bleeding.^8^, ^9^, ^10^ Consequently, it is highly needed to explore natural ingredients with less undesirable side effects, especially those of plant origin, that could substitute or alleviate the need for conventionally used drugs to prevent postmenopausal osteoporosis.

Traditional Chinese medicine (TCM) has been widely used for a long time to treat bone disease and will undoubtedly be a vast and yet largely untapped source to develop an appropriate powerful alternative to treat this disease. Many bioactive ‘natural’ products usually have less undesirable side effects in certain dose and have been discovered from traditional Chinese herb, such as genistein,^11^ 17β‐oestradiol and ginsenoside,^12^ which showed efficient anti‐osteoporosis effect. According to the theory of traditional Chinese medicine, the kidneys contain an ‘essence’ that regulate bone metabolism and generate marrow. Thus, kidney deficiency leading to bone loss is considered to be associated with the pathological process of postmenopausal osteoporosis.^13^, ^14^ As such, nourishing the kidney become one of basic fundamental for treating osteoporosis and commonly the administration of this kind of herbal medicines cause patients satisfactory efficacy. Rhizoma Drynariae (RD), the dried rhizome of *Drynaria fortunei* (Kunze) J. Sm., has been frequently used as a kidney‐tonifying and anti‐osteoporosis herb in traditional Chinese medicine to manage nephrasthenia syndrome and bone‐related diseases, such as bone fracture, osteoporosis and arthritis for thousands of years.^15^, ^16^, ^17^ Accumulating evidence has reported the osteoprotective effects of RD in both in vitro and in vivo studies,^18^ and phytochemical analysis of RD indicated that flavonoids 19, 20 and phenylpropanoids 21, 22 were the active substances responsible for its osteoprotective activities. However, up to now, there is no available information with regard to the isolation and characterization of the polysaccharide from this plant, let alone his bone‐protective effects. In this respect, the present study was conducted to isolate and characterized bioactive polysaccharide from this plant and investigate its effect on postmenopausal osteoporosis in ovariectomized (OVX) rats.

## 2 MATERIALS AND METHODS

### 2.1 Materials and reagents

The dried rhizome of *D fortunei* was purchased from Dongxingtang drug Market in Zhanjiang (China) and was cut into small pieces and then powdered by high‐speed disintegrator, following passed through a 60 mesh sieve. Standard molecular weights of T‐dextran (T‐2000, T‐500, T‐70, T‐40 and T‐4) and nine monosaccharides (glucose, arabinose, rhamnose, mannose, galactose, xylose and fucose) were obtained from Sigma Chemical Co. DEAE Sepharose fast flow and Sepharose CL‐6B were purchased from Amersham Pharmacia Co. (Sweden). Serum (S)‐Ca (20162400906), S‐P (20162400909), urinary (U)‐Ca (20162400904) and U‐P (20162400908) ELISA kit were purchased from Biosino Bio‐Technology and Science Inc. ALP (AF2910) and DPD/Cr (KGE005) ELISA kit were purchased from R&D systems. All other analytical chemical reagents were of analytical grade and were purchased from Peking Chemical Co.

### 2.2 Isolation and purification of polysaccharide from the rhizome of D fortunei

First, the dried rhizome of *D fortunei* (1000 g) was sliced, smashed and stored at room at room temperature before extraction. Next, the dried powder of *D fortunei* rhizome was extracted in turn with 80% ethanol and petroleum ether to eliminate interference impurity, such as pigments, lipids, low molecular weight compounds and disaccharides. The reaming residue was extracted with boiling water in a ratio of 1:8 (w/v) for 2.0 hours and repeated three times until the supernatant became colourless. The extraction mixture was combined and concentrated in a rotary evaporator under reduced pressure at 50°C, followed by centrifugation at 3000 g for 10 minutes. The resulting supernatants were precipitated by adding 95% (v/v) ethanol to a final concentration 80% (v/v) and kept at 4°C overnight. After centrifugation at 3000 g for at 4°C for 10 minutes, the precipitate was dissolved in distilled water and deproteinized using sevage method.^23^ The supernatants layers containing polysaccharide were collected, concentrated and then subjected to ethanol precipitation as described above to yield the crude polysaccharide (CDFP, 78 g), with a yield of 7.8% (w/w) of raw material.

Each aliquot of 100 mg of CDFP was redissolved in 5 mL distilled water and filtered through Whatman filter. Then, the filtrate was applied to a DEAE Sepharose Fast Flow column (5.0 × 50 cm), which was eluted successively with distilled water and different concentrations of stepwise NaCl solution (0.25, 0.5 and 1.0 mol/L) solutions for 300 minutes, respectively, at a flow rate of 2.0 mL/min. The eluates were collected in 8 mL per tube, and the carbohydrate content was determined by the phenol‐sulphuric acid method.^24^ Three peak fractions containing polysaccharides were pooled and named CDFPW (distilled water), CDFPA (0.25 mol/L NaCl), CDFPB (0.5 mol/L NaCl) and CDFPC (1.0 mol/L NaCl). CDFPW was further purified with a column of Sepharose CL‐6B (2.0 × 100 cm) by the difference in molecular sizes and eluted with distilled water at a flow rate of 0.5 mL/min. The relevant fractions were collected, concentrated and finally lyophilized to obtain one purified polysaccharide (DFPW, 5.5 g), with a yield of 0.55% (w/w) of raw material.

### 2.3 Chemical composition, monosaccharide composition and molecular weight analysis

The neutral sugar content of the polysaccharide was measured by the phenol‐sulphuric acid method using glucose as the standard.^24^ The protein content was performed according to the Bradford's method with a standard curve using bovine serum albumin (BSA).^25^ The uronic acid content was determined by the m‐hydroxydiphenyl method with D‐glucuronic acid as the standard.^26^

The monosaccharide composition was determined by gas chromatography (GC) (HP6890, Agilent Technologies) equipped with an Hp‐5MS capillary column (30 m × 0.25 mm × 0.25 μm) (Aglient Technologies) and flame ionization detector (FID) using the method in a previous study.^27^ Briefly, 2.0 mg of polysaccharide sample or standard monosaccharide was dissolved in 4 mol/L trifluoroacetate (2 mL) in a closed tube and then hydrolysed at 120°C for 2 hours. Excess acid was removed by co‐distillation under nitrogen with methanol and dried in a vacuum evaporator at 50°C. Then, the hydrolysate was dissolved in distilled water (100 μL) and reduced by NaBH_4_, followed by acetylation in pyridine‐acetic acid (200 μL; 1:1, v/v), for 30 min at 100°C. The resulting alditol acetate was extracted with CHCl_3_ and analysed by GC. The column temperature was programmed to rise from 110 to 210°C at a rate of 5°C/min and held at 210°C for 3 minutes, then programmed at a speed of 2°C/min to 250°C and held at 250°C for 3 minutes and finally programmed to 280°C at 10°C/min. Monosaccharide composition analysis was done by comparison with reference sugars (glucose, arabinose, rhamnose, mannose, galactose, xylose and fucose).

The molecular weight of the sample was detected using a Shimadzu LC‐2010A HPLC (Shimadzu Corp), which was equipped with a refractive‐index detector (RID‐10A, Shimadzu) and a TSK‐gel PWXL G5000 column (7.8 mm × 300 mm, Shodex). The injection volume was 20 μL for each run, and 0.7% Na_2_SO_4_ was used as the mobile phase at a flow rate of 0.8 mL/min. Series dextrans including T‐4 (molecular mass, 4 × 10^3^ Da), T‐10 (1 × 10^4^ Da), T‐40 (4 × 10^4^ Da), T‐70 (7 × 10^4^ Da), T‐500 (5 × 10^5^ Da) and T‐2000 (2 × 10^6^ Da) were used as the molecular mass markers to create the calibration curve. The molecular weight was calculated using Agilent GPC software by comparison with the calibration curve constructed above.

### 2.4 Animals and treatments

Female Sprague‐Dawley (SD) rats aged 3 months were obtained from the Experimental Animal Center of Guangdong Medical University and were allowed to acclimate to the new environment for a week in plastic cages at 22°C under 12‐hour light and 12‐hour dark cycle per day before the study was started. During the experimental period, all the rats were allowed free access to distilled water and fed with commercial food pellets. All animals were treated according to the Ethic Principles for Care and Use of Laboratory Animals with the approval of the Institutional Ethics Committee of Guangdong Medical University, China (REC: PJ2018037).

The acclimatized rats underwent either bilateral oophorectomy (OVX group, n = 60) or bilateral laparotomy (sham group, n = 12) under pentobarbital sodium (50 mg/kg bodyweight, ip) anaesthesia. After 4 weeks, the OVX rats were randomly divided into four groups: OVX with vehicle (OVX, n = 10); OVX with Raloxifene (RLX, 1 mg/kg bodyweight); low dose DFPW‐treated (DFPWL, n = 10, 100 mg/kg bodyweight/d) and high dose DFPW‐treated (DFPWH, n = 10, 400 mg/kg bodyweight/d). The DFPW sample was suspended in distilled water and given to mice via intragastric administration for additional 12 weeks. Rats in the sham and OVX control groups were orally administered with the same volume of distilled water. The bodyweight of the animals was recorded weekly during the experimental period.

### 2.5 Collection and preparation of serum, urine and bone sample

Before kill, rats were fasted for 12 hours in individual cage and the urine samples were collected in separators. After laparotomy using anaesthesia with diethyl ether, blood samples were taken from the abdominal aortae and serum was collected by centrifugation (4°C, 2000 g, 10 minutes). Urine and serum samples were then stored at −80°C until biochemical analysis. The femurs of both sides were isolated from adjacent connective tissues of dead rats, wrapped in saline‐soaked gauze bandages to prevent dehydration and stored at −20°C for further biomechanical testing and structural analysis.

### 2.6 Measurement of bone mineral density

The bone density was measured using dual‐energy X‐ray absorptiometry (DXA, Lunar DPXL, Software version 1.0C), and the results were expressed as mg/cm^2^ of surface area as described elsewhere.^28^, ^29^, ^30^

### 2.7 Measurement of serum, bone and urine biochemical markers analysis

The level of serum ALP, Ca and P; urine deoxypyridinoline (U‐DPD)/Cr, U‐Ca/Cr and U‐P/Cr; bone Ca, P and Mg were evaluated with respective ELISA kit according to the manufacturer's instructions.

### 2.8 Histopathological analysis

The morphology of the sample was examined as described in a previous report.^31^ In general, the fresh femur specimens were fixed in 10% phosphate buffered formalin for 36 hours at 4°C, decalcified in 5% ethylene diamine tetra acetic acid (EDTA) for 7 days and then embedded in paraffin wax. The block was cut into sections (4 mm thick) along the sagittal plane passing through the transversal axis of the femurs. Afterwards, the sections were stained with haematoxylin and eosin (H & E) for morphology examination under the optical microscope (Olympus Corporation).

### 2.9 Bone biomechanical measurement

Biomechanical testing was performed in the left femora tissue using a three‐point bending machine (Electro‐mechanical Material Testing Machine, Avalon Technologies) with length of 20 mm for two support points and a 1.5 mm/min loading speed to assess bone strength. The load was performed to the central femoral area, and the displacement at osteoid tissue collapse (fracture) (mm) and load (N) was recorded until the specimen was broken. The biomechanical parameters including the maximum load, energy absorption, maximum stress and Young's modulus were obtained based on the load‐deformation curve and calculated as previously described formulas.^32^

### 2.10 Statistical analysis

All values from these experiments are expressed as mean ±(SD) for each group and were subjected to One‐way analysis of variance (ANOVA) followed by Student's *t* test for comparison of two groups. *P* values of less than .05 were accepted as statistically significant.

## 3 RESULTS

### 3.1 Isolation and characterization of the polysaccharide DFPW from the rhizome of D fortunei

**Figure 1:**
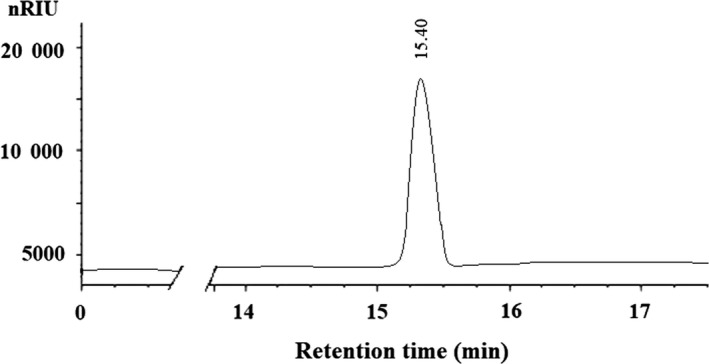
HPGPC profile of the polysaccharide DFPW

**Figure 2:**
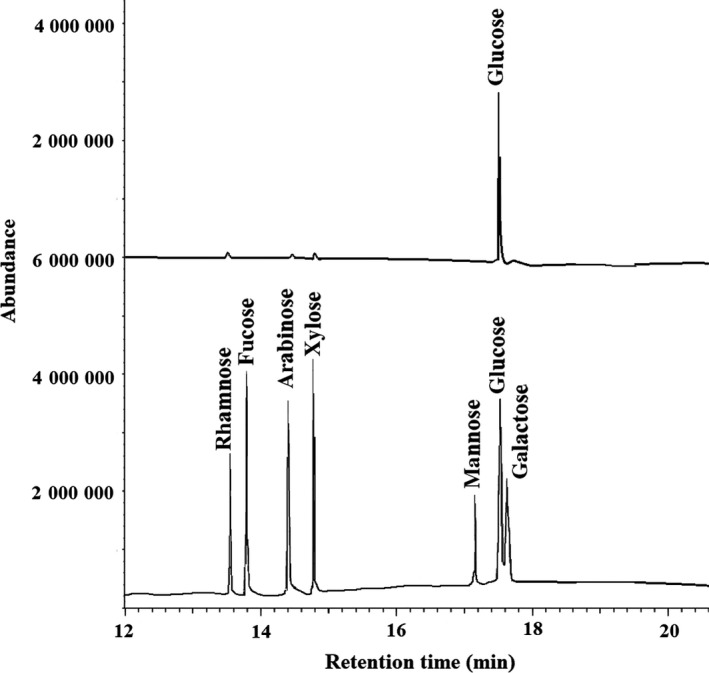
The GC chromatograms of the standard monosaccharides mixture (glucose, arabinose, rhamnose, mannose, galactose, xylose and fucose) and monosaccharide composition of DFPW

The crude polysaccharides CDFP were obtained from defatted rhizome of *D fortunei* by hot water extraction, ethanol precipitation, deproteinization and washed in turns with absolute ethanol, acetone and ether to dry the polysaccharide, with a yield of 7.80% of the starting raw material. CDFP was first stepwise separated using a column filled with DEAE Sepharose Fast Flow chromatogram and eluted with 0, 0.25, 0.5 and 1.0 mol/L NaCl solution to give four primary independent elution peaks (CDFPW, CDFPA, CDFPB and CDFPC), respectively. Their individual yield was 20.54%, 8.56%, 6.02% and 3.25% of the parent polysaccharide CDFP. To obtain high purity and production of polysaccharide for further study used, CDFPW was performed over a gel filtration chromatography on a Sepharose CL‐6B column to yield a purified water‐soluble polysaccharide DFPW, with a 0.55% yield of raw material. The phenol‐sulphuric acid assay showed that DFPW contained 96.87% of carbohydrates. No absorption at 280 and 260 nm in the UV spectrum suggested the absence of protein and nucleic acid, which was further confirmed by m‐hydroxydiphenyl and the Lowry method, indicating it is a neutral polysaccharide. The chromatogram of DFPW on HPSEC demonstrated a single and symmetrically sharp peak, confirming its homogeneity characterization (Figure 1). The average molecular weight of DFPW was determined to be 3.2 × 10^4^ Da, with a retention time of 15.40 minutes by comparison with the calibration curve made with standard dextrans (logMw = 7.908‐0.2213Rt, R = 0.9828, where Mw represents relative molecular weight, Rt represents retention time). Monosaccharide composition analysis by GC showed that DFPW was composed of only glucose (Figure 2).

### 3.2 The effect of the polysaccharide DFPW on the bodyweights of OVX rats

At the beginning of the animal experiments, fix groups of rats had a similar initial mean bodyweight and then the bodyweight of the rat increased continuously throughout the study in all groups (Table 1). Even though daily food consumption was not different in each group, by the end of the work, the mean bodyweight of the OVX group continued to be significantly higher than that of the sham group (*P* < .01), with 28.71% increase relative to initial weight, whereas only 7.56% significant increment was observed in RLX treated rats after 12 weeks, which was statistically different with that of OVX control (*P* < .01). At the same time, different doses of DFPW (100 or 400 mg/kg) administration resulted in significantly inhibitory effect on OVX‐induced bodyweight increase as compared with OVX group (*P* < .05), although the efficiency is not visibly higher than RLX treatment.

**Table 1:** Effects of DFPW on the bodyweights of OVX rats

Group	Bodyweight (g)			Increased rate relative to initial weight (%)
	Initial	Final	Difference	
Sham	255.51 ± 7.25	273.58 ± 7.68	18.07	7.07
OVX	252.67 ± 6.85	325.20 ± 12.02*	72.53**	28.71
RLX (1 mg/kg)	257.08 ± 7.21	276.52 ± 9.25^#^	19.44^##^	7.56
DFPWL	253.32 ± 8.02	283.21 ± 9.58^#^	29.89*^#^	11.80
DFPWH	255.45 ± 6.54	280.82 ± 9.13^#^	25.37*^#^	9.93

Note

All values are expressed as mean ± SD. *P < .05; **P < .01 vs Sham group; ^#^ *P* < .05; ^##^ *P* < .01 vs OVX group.

### 3.3 The effect of the polysaccharide DFPW on the bone histology and morphology in OVX rats

**Figure 3:**
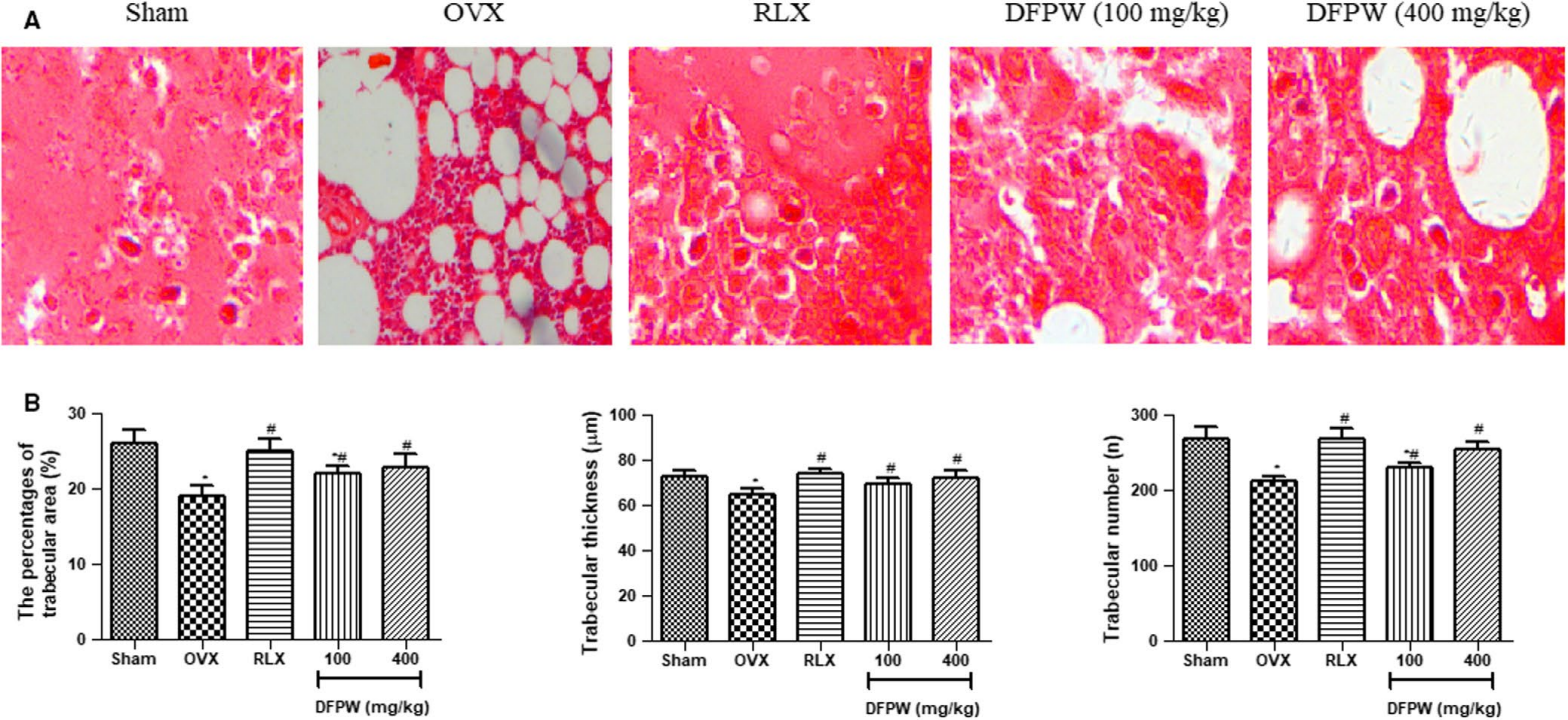
DFPW rescued the OVX‐induced bone loss of femur in rats. A, Photomicrograph of femur section of OVX rats stained with haematoxylin and eosin; B, ffects of DFPW or RLX on the percentages of trabecular area, trabecular thickness and number in OVX rats. All values are expressed as mean ± SD ^*^ *P* < .05 vs Sham group; ^#^ *P* < .05 vs OVX group

The OVX female rat model was used to assess the anti‐osteoporosis activity of DFPW. The histopathological studies were carried out on sections of femur specimens by haematoxylin and eosin staining and observed under microscope for the microarchitectural changes. As seen in Figure 3A and 3, the percentages of trabecular area, trabecular thickness and number in OVX rats decreased and was considerably lower than those in the normal rats (*P* < .05). However, in the presence of RLX or DFPW, the change in these indexes on bone histology and morphology was all recovered and even normalized, and effects were statistically significant in comparison with the OVX control group (*P* < .05). This indicated that DFPW was effective in eliminating the negative effects of OVX on the trabecular bone mass.

### 3.4 The effect of the polysaccharide DFPW on bone mineral density and bone mineral content in OVX rats

As shown in Table 2, femur bone density and tibia bone density dropped obviously compared with the sham group (from 0.20 ± 0.03 in sham control to 0.13 ± 0.02 mg/cm^2^ in OVX rats for femur and from 0.16 ± 0.03 in sham control to 0.10 ± 0.02 mg/cm^2^ in OVX rats for tibia). In a same period of time, treatments of DFPW at a dosage of 100 or 400 mg/kg appreciably recovered the loss of bone density to 0.17 ± 0.03 mg/cm^2^ (*P* < .05) and 0.18 ± 0.03 mg/cm^2^ (*P* < .01) for femur bone density and 0.14 ± 0.02 mg/cm^2^ (*P* < .05) and 0.16 ± 0.02 mg/cm^2^ (*P* < .05) for tibia bone density, respectively, in comparison with 0.13 ± 0.02 mg/cm^2^ (femur) or 0.10 ± 0.02 mg/cm^2^ (tibia) of the model group, which approximated to the level of RLX treatment. Furthermore, we examined the beneficial effects of DFPW treatments on femur bone mineral components including Ca, P and Mg. All three bone mineral in all five experimental groups showed a similar tendency as mineral density of femur (Table 2). The content of Ca, P and Mg of femur in the OVX animals was also statistically significant from sham control after 12 weeks (*P* < .01), but after treatments with RLX or DFPW (100 or 400 mg/kg), the significant improvement was observed for these parameters with relative to those in the OVX rats. This result demonstrated that DFPW could prevent the progress of bone loss induced by OVX.

**Table 2:** Effects of DFPW on bone mineral density and bone mineral content in OVX rats

Group	Bone mineral density (mg/cm^2^)		Bone mineral content (mmol/g)		
	Femur bone	Tibia bone	Ca	P	Mg
Sham	0.20 ± 0.03	0.16 ± 0.03	4.96 ± 0.42	2.88 ± 0.25	0.31 ± 0.05
OVX	0.13 ± 0.02**	0.10 ± 0.02*	4.12 ± 0.38**	2.22 ± 0.21**	0.23 ± 0.03**
RLX (1 mg/kg)	0.19 ± 0.03^##^	0.17 ± 0.03^##^	4.88 ± 0.41^##^	2.78 ± 0.23^#^	0.32 ± 0.04^##^
DFPWL	0.17 ± 0.03^#^	0.14 ± 0.02^#^	4.49 ± 0.43*^,#^	2.66 ± 0.22*^,#^	0.29 ± 0.04^#^
DFPWH	0.18 ± 0.03^##^	0.16 ± 0.02^#^	5.05 ± 0.44^#^	2.89 ± 0.26^#^	0.33 ± 0.05^#^

Note

All values are expressed as mean ± SD. *P < .05, **P < .01 vs Sham group; ^#^ *P* < .05, ^##^ *P* < .01 vs OVX group.

### 3.5 The effect of the polysaccharide DFPW on serum and urinary biochemical markers in OVX rats

The results of serum and urine biochemical parameters in all groups were shown in Table 3. In the present study, OVX rats showed a remarkable increased ALP activity compared with that in rats of the sham group (*P* < .01), suggesting that OVX inhibits bone formation. In contrast, RLX or DFPW (100 or 400 mg/kg) treatment restored the increased levels of ALP, suggesting its beneficial effect in bone remodelling. After twelve weeks of treatment, the results indicated a significant increase in the urinary DPD/Cr level in the OVX group when compared with the sham group (*P* < .01). The addition of RLX or DFPW (100 or 400 mg/kg) significantly suppressed the OVX‐induced increases in urinary DPD/Cr levels (*P* < .05 or *P* < .01), indicating that the DFPW can inhibit bone resorption. OVX increased U‐Ca/Cr and U‐P/Cr (*P* < .01 for both) excretion when compared with the sham group, but RLX or DFPW (100 or 400 mg/kg) treatment could reverse the increase in both indices levels induced by OVX in a manner similar to DPD/Cr (*P* < .05 or *P* < .01). However, no remarkable changes were observed for S‐Ca and S‐P in all five groups. This data indicated that DFPW could improve bone mass by enhancing bone formation and inhibiting bone resorption.

**Table 3:** Effects of DFPW on biochemical parameters in serum and urine of OVX rats

Parameter	SHAM	OVX	RLX	DFPWL	DFPWLH
ALP (U/L)	112 ± 10.21	235 ± 19.21^**^	132 ± 11.20^**##^	145 ± 12.34^**##^	136 ± 11.23^**#^
S‐Ca (mmol/L)	2.77 ± 0.28	2.73 ± 0.29	2.76 ± 0.23	2.66 ± 0.21	2.72 ± 0.22
S‐P (mmol/L)	2.55 ± 0.28	2.60 ± 0.30	2.54 ± 0.27	2.62 ± 0.31	2.53 ± 0.29
DPD/Cr (nmol/mmol)	50.17 ± 4.77	88.71 ± 7.89^**^	60.13 ± 5.75^**##^	70.21 ± 6.22^**#^	63.31 ± 5.61^*#^
U‐Ca/Cr (mmol/mmol)	0.31 ± 0.04	0.78 ± 0.07^**^	0.48 ± 0.04^*#^	0.43 ± 0.05^*#^	0.35 ± 0.04^##^
U‐P/Cr (mmol/mmol)	3.02 ± 0.35	5.15 ± 0.63^**^	3.68 ± 0.39^##^	3.89 ± 0.47^*##^	3.21 ± 0.41^##^

Note

All values are expressed as mean ± SD. ^*^ *P* < .05, ^**^ *P* < .01 vs Sham group; ^#^ *P* < .05, ^##^ *P* < .01 vs OVX group.

### 3.6 The effect of the polysaccharide DFPW on biomechanical quality of the femur

The ability of DFPW to improve biomechanical parameters of femoral diaphysis in OVX rats was performed using three‐point bending tests. As mentioned in Table 4, OVX resulted in a significant decrease in maximum load, energy, Young's modulus and maximum stress in rats as compare with the sham group (*P* < .01). By the treatment of RLX or DFPW (100 or 400 mg/kg), maximum load and energy could be obviously improved compared with the OVX group (*P* < .05 or *P* < .01). Meanwhile, RLX or DFPW (100 or 400 mg/kg) treatment significantly reversed the decrease in Young's modulus and maximum stress in OVX rats (*P* < .05 or *P* < .01). Bone strength is one of the major characteristics of bone health,^47^ and DFPW could strengthen the hardness and strength, which might be associated with the stimulatory activity for bones based on the above results.

**Table 4:** Effects of DFPW on bone biomechanical parameters in OVX rats

Parameter	SHAM	OVX	RLX	DFPWL	DFPWH
Maximum load (N)	135.54 ± 10.52	100.35 ± 9.52^**^	129.65 ± 11.25^#^	115.79 ± 10.91 ^*#^	120.92 ± 10.32 ^*#^
Energy (N × mm)	50.36 ± 5.32	41.39 ± 4.52^**^	49.85 ± 5.87^#^	46.21 ± 5.05^#^	49.46 ± 5.21^##^
Young's modulus (MPa)	69 254.87 ± 720.354	4100.56 ± 400.68^**^	6755.64 ± 659.54^##^	5846.45 ± 569.87^*##^	6154.37 ± 628.64^*##^
Maximum stress (MPa)	186.67 ± 16.82	146.38 ± 13.65^**^	179.82 ± 15.88^*#^	165.71 ± 15.54^*#^	170.54 ± 15.21^*#^

Note

All values are expressed as mean ± SD. ^*^ *P* < .05, ^**^ *P* < .01 vs Sham group; ^#^ *P* < .05, ^##^ *P* < .01 vs OVX group.

## 4 DISCUSSION

In the past decades, some bioactive polysaccharides have attracted much research attention owing to their wide range of biological activities, such as anti‐viral,^33^ anti‐inflammatory,^34^ antioxidant,^35^ anti‐ulcer,^36^ immunostimulatory,^37^ antitumor activity and so on.^38^ It is also worth mentioning that these natural polysaccharides have been recognized as a source of safe and potent natural medicine because of their lower toxicity and higher efficacy.^39^ In recent years, several polysaccharides isolated from TCM, such as *Achyranthes bidentata*,^40^ Dipsacus asper 41 and Epimedium brevicornum,^42^ exhibited more significant anti‐osteoporotic effect in vitro or in vivo. However up to now, there are still no available report concerning the study on the isolation and anti‐osteoporosis activity of polysaccharide from *D fortune*i, a popular TCM used to treated bone disorders in ancient time of China. In this work, we successfully isolated and purified one homogenous polysaccharide DFPW from defatted rhizome of *D fortunei*. DFPW contained glucose as dominating monosaccharide and its average molecular weight was estimated to be 3.2 × 10^4^ Da. The objective of this study was to evaluate the anti‐osteoporosis activity of DFPW in rats and discover the underlying mechanisms.

Ovariectomy often causes trabecular bone loss in rats; therefore, OVX rats are widely used as a classical and reliable model in postmenopausal osteoporosis.^43^, ^44^ In present study, an OVX‐induced rat model was used to evaluate the efficacy of DFPW in postmenopausal osteoporosis. Following 12 weeks treatment, although no statistically significant difference in baseline weights of all groups, all the groups had significantly higher bodyweight gain compared with their initial bodyweights after 12 weeks of treatment. The OVX rats have significantly higher bodyweights than that of sham‐operated rats due to fat deposition caused by oestrogen deficiency,^45^ and this bodyweight gain was totally inhibited by RLX or DFPW (100 or 400 mg/kg) treatment. This observing suggested that DFPW at these dosages behaved like RLX in the regulation of bodyweight in the OVX rats.

Keeping trabecular microarchitecture in its original state or in good condition is important in maintaining bone strength, which may reduced the risk of fracture.^46^ Therefore, we then examined the effect of DFPW treatment on histological change OVX rats by visualizing and quantifying the bone trabecular structure including trabecular area percentage, thickness and its number with haematoxylin and eosin staining methods. Under the light microscope, haematoxylin and eosin staining of the femurs in OVX rats showed an aggravated disrupted microarchitecture as compared with sham control, as determined by the decrease in the percentages of trabecular area, trabecular thickness and its number. In contrast, these abnormal histological changes were almost restored in the rats receiving RLX or DFPW (100 or 400 mg/kg) treatment, suggesting the protective effect of DFPW on bone loss of the total femur in OVX rats.

Bone mineral density and bone mineral content are considered to be the classic standards to measure the incidence of osteoporosis.^47^, ^48^ Significant decrease of bone mineral density in both femur and tibia bones was observed in OVX rats, implying that OVX induced a typical osteoporosis. The 12‐week treatment with RLX or DFPW (100 or 400 mg/kg) significantly increased the bone mineral density of femur and tibia bones. Similarly, OVX‐induced loss of bone mineral contents (Ca, P and Mg) was particularly reversed by RLX or DFPW (100 or 400 mg/kg). This preventive effect of DFPW on bone mineral loss of OVX rats was comparable to other active ingredients from natural plant for the treatment of osteoporosis.^11^, ^49^

In osteoporosis, biochemical markers of bone turnover have been used as a popular tool to examine the effects of different drugs on bone formation.^50^, ^51^ ALP activity, an important biochemical marker of bone formation, was increased in many bone metabolic disorders including osteoporosis due to increased bone turn over.^52^ DPD is widely distributed in bone and dentin,^53^ and its level in urine is regarded as a specific marker for bone resorption, which have been documented in studies of postmenopausal women and laboratory animals.^54^, ^55^ Furthermore, fasting U‐Ca/Cr and U‐P/Cr level are also the commonly used useful variable for estimating net bone resorption.^56^ At the end of the study, the ALP activity and urinary DPD/Cr level were found to be increased in OVX rats, corresponding to reduced bone formation and increased bone resorption, respectively. Treatment with DFPW (100 or 400 mg/kg) or RLX to OVX rats was able to return both marker to approach the level as seen in the sham‐operated group. Seemly, increased U‐Ca/Cr and U‐P/Cr excretion induced by OVX were dramatically prevented in rats suffering DFPW (100 or 400 mg/kg) or RLX treatment. Additionally, OVX rats showed unchanged levels of S‐Ca and S‐P levels, indicating that balanced mineral homeostasis and lesser bone mineralization despite OVX, which was similar with the other reports for S‐Ca and S‐P levels in OVX rats.^44^, ^57^ It was likely inferred that bone mass maintained in OVX rats by DFPW administration was due to enhancing bone formation and inhibiting bone resorption.

There is abundant evidence suggesting that OVX not only leads to a decrease in bone density, but also in biomechanical strength of bones.^57^ We next evaluated the effect of DFPW on biomechanical parameters of femoral diaphysis in OVX rats using three‐point bending tests. The same observation was observed in OVX rats, as demonstrated by the decrease in maximum load, energy, Young's modulus and maximum stress in femoral diaphysis when compared with the sham group. All these deterioration was reversed to the opposite in OVX rats in response to RLX or DFPW (100 or 400 mg/kg) administration. Bone strength is one of the major characteristics of bone health,^58^ and DFPW could strengthen the hardness and strength of bone, which might be attributable to the prevention and treatment of postmenopausal osteoporosis.

## 5 CONCLUSIONS

Based on the effects of DFPW on the bone markers of OVX rats, it is likely concluded that daily DFPW oral administration would be a useful remedy for treating postmenopausal osteoporosis in rats, comparable to RLX. This function was related to the regulation of DFPW on the balance between bone formation and bone resorption, as such, the bone loss could be relieved in OVX rats. This investigation shed the light on the ongoing experiment to discover if this polysaccharide DFPW would take into effect for the prevention and treatment of postmenopausal osteoporosis for human. Further studies are required to elucidate its anti‐osteoporotic mechanisms, and we believe that DFPW can be developed as a potential anti‐osteoporotic therapeutic.

## CONFLICT OF INTEREST

The authors declare that they have no competing interests.

## AUTHOR CONTRIBUTIONS

XS and JS designed the experiment and interpreted results. XS and BW drafted manuscript. BW, ZP, XC, QF, CW and JZ performed experiments. All authors have read and approved the final manuscript. XS is corresponding author.

## Data Availability

All data generated or analysed during this study are included in this article.
